# Modulation of the immune response by the *Pseudomonas aeruginosa* type-III secretion system

**DOI:** 10.3389/fcimb.2022.1064010

**Published:** 2022-11-28

**Authors:** Albane Jouault, Alessandra Mattos Saliba, Lhousseine Touqui

**Affiliations:** ^1^ Mucoviscidose: Phénotypique et Phénogénomique, Centre de Recherche Saint-Antoine, Sorbonne Universités, UPMC Univ Paris 06, INSERM, Paris, France; ^2^ Département Santé Globale, Mucoviscidose et Bronchopathie Chroniques, Institut Pasteur, Paris, France; ^3^ Department of Microbiology, Immunology and Parasitology, Faculty of Medical Sciences, Rio de Janeiro State University, Rio de Janeiro, Brazil

**Keywords:** type-III secretion system, *Pseudomonas aeruginosa*, exotoxins, inflammation, pathogenicity

## Abstract

*Pseudomonas aeruginosa* is an opportunistic pathogen that can cause critical cellular damage and subvert the immune response to promote its survival. Among the numerous virulence factors of *P. aeruginosa*, the type III secretion system (T3SS) is involved in host cell pathogenicity. Using a needle-like structure, T3SS detects eukaryotic cells and injects toxins directly into their cytosol, thus highlighting its ability to interfere with the host immune response. In this mini-review, we discuss how the T3SS and bacterial effectors secreted by this pathway not only activate the immune response but can also manipulate it to promote the establishment of *P. aeruginosa* infections.

## Introduction

*Pseudomonas aeruginosa* is a Gram-negative bacterium causing infections in immunocompromised individuals. This pathogen is one of the ESKAPE pathogens (including *Enterococcus faecium*, *Staphylococcus aureus*, *Klebsiella pneumoniae*, *Acinetobacter baumanii*, *P. aeruginosa*, *Enterobacter* spp.), which constitute life-threatening nosocomial bacteria (Hirsch and Tam, 2010; Mulani et al., 2019). *P. aeruginosa* also infects patients with specific pathologies such as cystic fibrosis (CF). Due to its ability to form a biofilm, *P. aeruginosa* often chronically infects CF patients and represents a negative outcome in this disease (Malhotra et al., 2019).

To successfully establish itself in the host, *P. aeruginosa* deploys a series of virulence factors, including toxins, siderophores, adhesins, and secretion systems (see reviews of Gonçalves-de-Albuquerque et al., 2016; Qin et al., 2022). The latter allows the transport of molecules into the extracellular media or host cells. Among the known secretion systems of *P. aeruginosa*, the type III secretion system (T3SS) is the most relevant in human pathogenesis and is implicated in host invasion by injecting toxins directly into eukaryotic cells (Hauser, 2009; Juan et al., 2017). It plays a significant role in the colonization of the host by *P. aeruginosa*, and several studies show a close relationship between T3SS expression and the modulation of the host immune system. This review aims to discuss the current knowledge regarding the interactions between T3SS and the host immune response.

## T3SS structure

The *P. aeruginosa* T3SS is a complex machinery that includes a needle complex, a translocon, effectors, chaperones, and a regulatory system (reviewed by Hauser, 2009; Horna and Ruiz, 2021).

**Figure 1:**
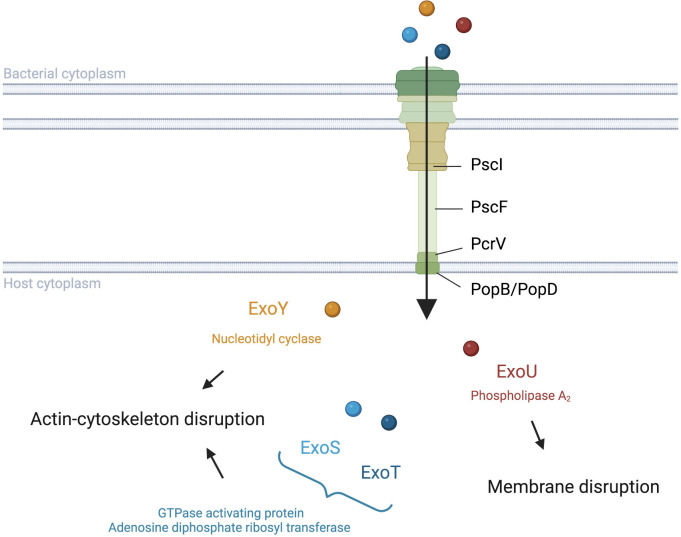
Translocation and activities of *Pseudomonas aeruginosa* T3SS effectors into host cells. *P. aeruginosa* injects exotoxins though a needle complex after contact with the surface of the targeted eukaryotic cell. ExoS and ExoT modulate the cytoskeleton with their GAP and ADPRT domains (Barbieri and Sun, 2005). ExoY also impacts the actin cytoskeleton but with its Nucleotidyl Cyclase activity (Belyy et al., 2018). ExoU disrupts the integrity of the lipid membrane *via* its PLA2 activity (Schulert et al., 2003; Sato and Frank, 2004).

The needle complex consists of a multi-ring base and a needle-like filament (**Figure 1**). The multi-ring base includes the PscC protein for the outer rings and the PscD and PscJ proteins for the inner ring (Notti and Stebbins, 2016). PscI connects the multi-ring base to the needle (Deng et al., 2017). The needle like-filament, composed of PscF subunits, allows the passage of effectors and serves as a sensor for host cell contact (Lombardi et al., 2019). The translocon, composed of the PopB, PopD, and PcrV proteins, is also reported as the needle tip complex. PcrV is involved in the assembly of the PopB/D complex, which allows the injection of effectors into host cells by forming a pore in the host cell membrane (Goure et al., 2004; Romano et al., 2011). As such, the pore formed can lead to host cell death regardless of effector action (Audia et al., 2013).

Although PemA, PemB, and the nucleoside diphosphate kinase have also been proposed as T3SS effectors (Neeld et al., 2014; Burstein et al., 2015), the four classically cited *P. aeruginosa* T3SS effectors are ExoS, ExoU, ExoT, and ExoY (**Figure 1**). The latter two toxins are detected with a high prevalence in contrast to ExoS and ExoU, which have been reported to be mutually excluded in most studies (Feltman et al., 2001; Ozer et al., 2019). ExoS and ExoT are two homologous bifunctional enzymes with GTPase activating protein (GAP) activity in the N-terminal region and adenosine diphosphate ribosyl transferase (ADPRT) activity in the C-terminal region (reviewed by Barbieri and Sun, 2005). GAP activity, targeting an array of GTPases, reorganizes the actin cytoskeleton, leading to cell rounding and disruption of cell-to-cell adhesion while ADPRT activity, targeting the Ras protein, modifies the cytoskeleton (Garrity-Ryan et al., 2000; Garrity-Ryan et al., 2004; Sun and Barbieri, 2004). Furthermore, both GAP and ADPRT domains induce apoptosis (Kaufman et al., 2000; Shafikhani et al., 2008; Wood et al., 2015; Kaminski et al., 2018). Although ExoS and ExoT possess similar domains, Shafikhani et al. (2008) suggest that the activity kinetic of these toxins could be different. ExoU is known to play a key role in the cytotoxic phenotype of *P. aeruginosa* through its phospholipase A2 (PLA2) activity (Sato et al., 2003; Schulert et al., 2003; Sato and Frank, 2004). This PLA2 activity, which produces lysophospholipids through the hydrolysis of membrane phospholipids, allows ExoU to disrupt the plasma membrane of host cells and cause rapid cell death (Phillips et al., 2003; Diaz and Hauser, 2010). ExoY is an actin-activated nucleotidyl cyclase that impacts the actin cytoskeleton (Yahr et al., 1998; Beckert et al., 2014; reviewed by Belyy et al., 2018). The actual clinical relevance of ExoY is still unclear, although a recent study showed a potential protective role of ExoY towards the cytotoxic effects of other *P. aeruginosa* virulence factors (Silistre et al., 2021).

Some effectors and other proteins implicated in T3SS need specific chaperones to facilitate their storage, conformational folding, and proper delivery to the secretion apparatus (see reviews Hauser, 2009; Horna and Ruiz, 2021).

The regulation of T3SS is complex and involves a variety of players. ExsA is the general transcriptional regulator binding promoter of T3SS genes, including its own promoter. Three additional proteins, ExsC, ExsD, and ExsE, control ExsA activity through a “catch and release” mechanism depending on whether *P. aeruginosa* is in contact or not with host cells (Hauser, 2009). Other players are also implicated in the regulation of T3SS transcription (For more details, see reviews Hauser, 2009; Horna and Ruiz, 2021).

## Implication of T3SS in the establishment of the IL-1β-mediated-inflammatory response to *P. aeruginosa*

**Figure 2:**
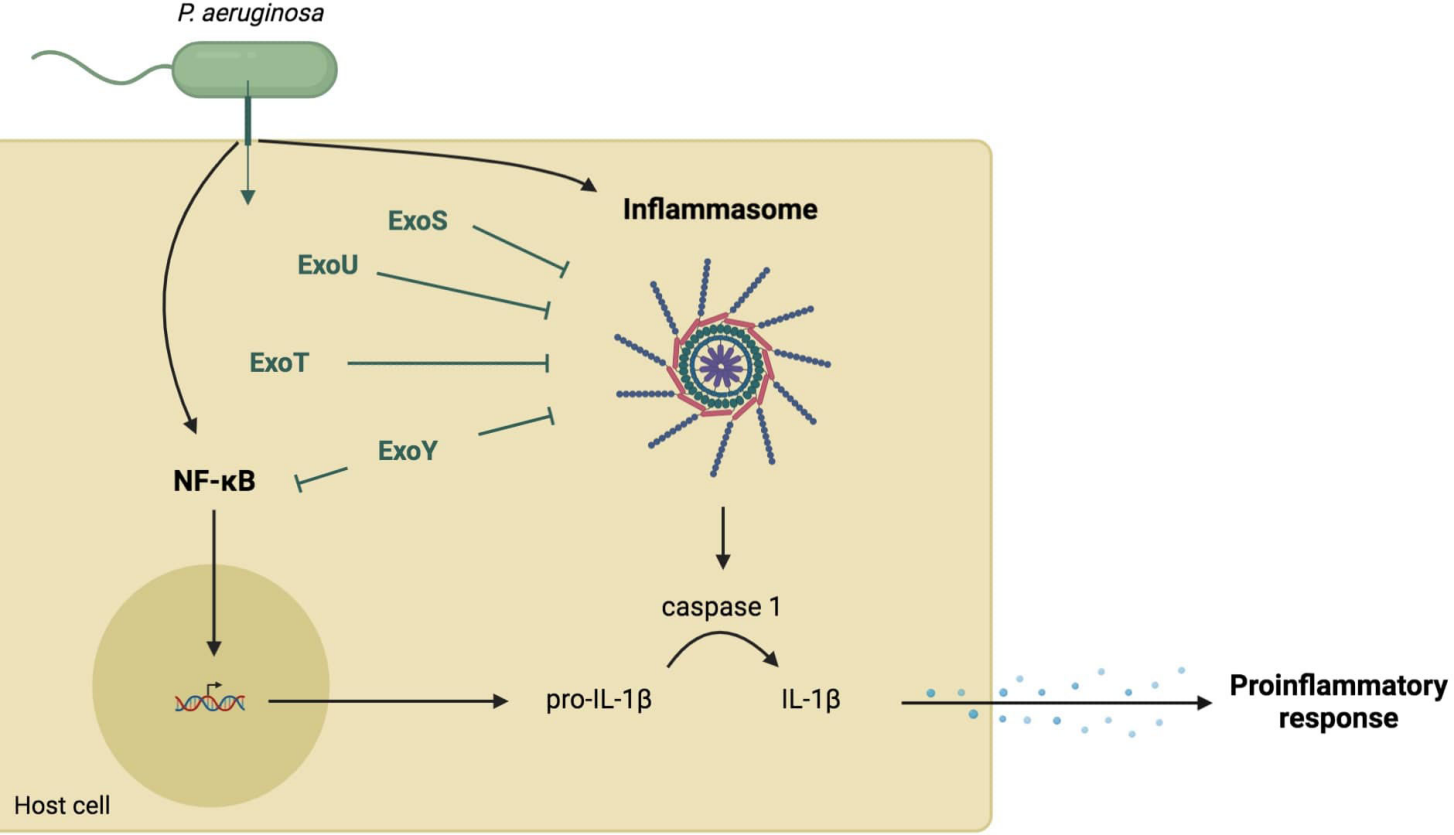
Role of T3SS in the cytokine IL-1β-dependent-proinflammatory response. Several components of the needle structure and the translocation apparatus activate the NF-κB pathway leading to the expression of pro-IL-1β (Grandjean et al., 2017; Yu et al., 2020). They also activate the inflammasome pathway resulting in a caspase-1-dependent maturation of IL-1β (Grandjean et al., 2017; Dortet et al., 2018). This cytokine production allows the recruitment of immune cells. However, injected exotoxins (in green) can inhibit both NF-κB and inflammasome pathways through various mechanisms.

The three translocon proteins of the needle tip, PcrV, PopB, and PopD, are required for *P. aeruginosa* to elicit rapid neutrophil recruitment into the airways, suggesting that T3SS affects the initial immune response of the host (Wangdi et al., 2010) to *P. aeruginosa* infection either directly, through the needle, or indirectly, through injection of exotoxins into the host cell. More specifically, T3SS can modulate the production of IL-1β whose signaling plays an important role in rapid neutrophil recruitment (**Figure 2**).

### NF-κB signaling pathway

The inducible transcription factor Nuclear Factor κB (NF-κB) regulates the transcription of genes involved in the immune and inflammatory responses. The binding of Pathogen-Associated Molecular Patterns (PAMPs) or Damage-Associated Molecular Patterns (DAMPs) to specific cell Toll-Like Receptors (TLR) leads to the activation of NF-κB signaling which ultimately results in the expression of proinflammatory genes including cytokines, chemokines, and other inflammatory mediators (reviewed by Liu et al., 2017). This process plays a key role in the initiation of immune and inflammatory responses.

Several components of the T3SS structure have been reported to activate the NF-κB signaling pathway. The T3SS inner-rod protein PscI and needle protein PscF are recognized by TLR4 and TLR2 (Grandjean et al., 2017), which can induce NF-kB activation. More importantly, the needle-tip protein PcrV seems to be a potent inducer of the NF-κB-mediated proinflammatory response (Yu et al., 2020) since the addition of PcrV on biofilm-infected tissues induces an inflammatory response. The latter was characterized by an increased macrophage differentiation toward an M1 phenotype followed by activation of the M1-polarized macrophages and phagocytosis, which occurred *via* the mitogen-activated protein kinases (MAPKs) and NF-κB signaling pathways. The authors of this study also reported downregulation of *pcrV* gene expression during *P. aeruginosa* biofilm infection, which was associated with attenuated immune activation. Altogether, these findings suggest that *P. aeruginosa* regulates T3SS to promote biofilm and its persistence in the host.

In addition to the needle structure, the ExoU toxin can also activate the proinflammatory response through the NF-κB signaling pathway. ExoU is involved in the inflammatory response through the generation of lysophospholipids and free arachidonic acid (via its PLA2 activity on host membrane phospholipids), at local sites of *P. aeruginosa* infection (Saliba et al., 2005). Lysophospholipids can be acetylated to generate Platelet-Activating Factor (PAF), an inflammatory lipid mediator that initiates neutrophil recruitment. By binding to its receptor, PAFR, located on airway epithelial cells, PAF activates NF-kB and stimulates IL-8 secretion (Mallet de Lima et al., 2014). Additionally, free arachidonic acid is also involved in ExoU-induced inflammation through its conversion into eicosanoids, including prostaglandin (PGE_2_ or PGI_2_) (Saliba et al., 2005), which are potent inducers of IL-6 and IL-8 production (Cho et al., 2014; Kawahara et al., 2015).

ExoY has been implicated in the decrease of IL-1β production and proinflammatory response both *in vitro* and in an animal model of lung infection by *P. aeruginosa* (Jeon et al., 2017; He et al., 2017; Kloth et al., 2018). Due to its adenylate cyclase activity, ExoY can reduce inflammasome-related responses by delaying the activation of NF-κB and caspase-1, resulting in a delayed inflammatory response (Jeon et al., 2017). Another study using a mouse model confirmed that ExoY can attenuate proinflammatory cytokine production by downregulating the activation of Transforming growth factor Activated Kinase 1 (TAK1), NF-kB, and MAPKs kinases (He et al., 2017).

### Inflammasome

NLRC4, as part of the Nod-like receptors (NLRs), allows host cells to sense pathogens and drive the innate immune response. PAMPs cause oligomerization and activation of NLRC4 inflammasome resulting in caspase-1-dependent maturation of IL-1β and IL-18 cytokines and pyroptosis. This cytokine production results in the recruitment of inflammatory leukocytes, such as neutrophils and monocytes/macrophages to the site of infection to achieve *P. aeruginosa* killing (reviewed by Broz and Dixit, 2016).

The T3SS inner-rod protein PscI and the needle protein PscF are recognized by macrophages through the neuronal apoptosis inhibitory protein (NAIP) family, which then leads to NLRC4 inflammasome activation (Yang et al., 2013; Grandjean et al., 2017). The translocation apparatus, with PopD, PopB, and PcrV proteins, has also been reported to induce IL-1β production (Sutterwala et al., 2007; Franchi et al., 2007; Miao et al., 2008; Galle et al., 2012). Moreover, one study showed that the pore-forming activity of PopD-PopB results in potassium efflux and histone H3 modifications. The authors suggested that this phenomenon could activate the inflammasome and subsequent IL-1β maturation (Dortet et al., 2018). However, another study reported that PopB activates the NLRP3 inflammasome rather than the NLRC4 inflammasome, similarly to pore-forming toxins from other organisms (Grandjean et al., 2017).

A recent study demonstrated that recognition of *P. aeruginosa* T3SS leads to an NLRC4 inflammasome response, limiting the development of infection in wounds. In response to T3SS insertion into bone marrow-derived macrophages, CrkII interacts with the Abl tyrosine kinase and enables the subsequent phosphorylation cascade through Abl → PKCδ → NLRC4, which is required for NLRC4 inflammasome assembly and activity (Mohamed et al., 2022). T3SS has also been reported to promote NLRC4 assembly and activation by inducing mitochondrial DNA and ROS release. Thus, the removal of damaged mitochondria generated after *P. aeruginosa* infection with functional T3SS blocks NLRC4 activation (Jabir et al., 2015). The flagellin FliC, which is a potent inducer of the NLCR4 inflammasome pathway, has also been reported to be translocated into host cells through T3SS and induces caspase-1 production (Ince et al., 2015).

Unlike the needle complex and the translocon, the *P. aeruginosa* T3SS effectors are implicated in NLRC4 dysregulation. ExoU was the first exotoxin shown to disrupt NLRC4 inflammasome activation, which transiently paralyzed the NLRC4 inflammasome (Sutterwala et al., 2007). Although the mechanisms involved still need to be elucidated, Hardy et al. (2022) recently showed an association between ExoU, host mitochondria, and caspase-1 activation. In addition to ExoU, ExoS has also been reported to regulate caspase-1-mediated IL-1β production by a mechanism dependent on its ADPRT activity (Galle et al., 2008). Recently, ExoT has been shown to inhibit NLRC4 inflammasome activation by disrupting CrkII/AbI interaction and blocking the phosphorylation cascade needed for NLRC4 assembly and function, resulting in a decrease in the inflammatory response (Mohamed et al., 2022).

Ince et al. (2015) showed that the ΔSTY mutant increases IL-1 release compared to the WT strain, suggesting a role of these toxins in the modulation of the IL-1 production pathway, as cited above. Interestingly, this inhibition is lost in the WT strain with an overexpression of FliC, suggesting that the expression of the agonists and antagonists of inflammasome activation could be controlled during specific steps of infection. This hypothesis could explain why studies on the benefit of inflammasome activation in the process of *P. aeruginosa* clearance is discussed and give an interesting perspective for further studies. (Schultz et al., 2003; Sutterwala et al., 2007; Franchi et al., 2007; Cohen and Prince, 2013; Faure et al., 2014; Mohamed et al., 2022).

## Subversion of host immune response by the *P. aeruginosa* T3SS

Activation of the host immune response by T3SS results in the killing of *P. aeruginosa* but the pathogen can solve this dilemma by deploying exotoxins to disrupt the response initiated by the host, which helps *P. aeruginosa* to avoid its phagocytosis. Not only does *P. aeruginosa* protect itself from the host response, it also eliminates other pathogens such as *S. aureus* from the airways by manipulating the host immunity.

### Antiphagocytosis

Some of the exotoxins injected by the *P. aeruginosa* T3SS into host cytosol can intoxicate immune cells or inhibit *P. aeruginosa* phagocytosis to favor its persistence in host tissues. Due to its PLA2 activity that hydrolyzes membrane phospholipids and promotes necrosis, ExoU has been shown to intoxicate and kill neutrophils, as well as other phagocytic cells (Diaz et al., 2008; Diaz and Hauser, 2010). Moreover, both ExoS and ExoT trigger neutrophil apoptosis *via* a mechanism mediated by the ADPRT domain (Sun et al., 2012). Besides directly injuring phagocytic cells, the ADPRT activity of ExoS also inhibits *P. aeruginosa* phagocytic uptake by neutrophils and macrophages (Rangel et al., 2014). Additionally, the GAP domain of ExoT and ExoS can also reduce the ability of host cells to phagocytize *P. aeruginosa* (Garrity-Ryan et al., 2000; Barbieri and Sun, 2005).

Although *P. aeruginosa* can kill immune cells extracellularly and avoid its phagocytosis, it was reported to be engulfed by phagocytes in animal and cell culture models (Garai et al., 2019). Once in contact with immune and epithelial cells, *P. aeruginosa* must evade cellular defense mechanisms against pathogens to survive intracellularly. Studies have suggested a key role for ExoS in the intracellular persistence of *P. aeruginosa*. In macrophages, ExoS has been reported to modulate phagocytic vacuole escape *via* a mechanism controlled by MgtC and OprF and involving the GAP activity of ExoS (Garai et al., 2019). On the other hand, the ADPRT activity of ExoS promotes bacterial survival in epithelial cells by establishing a protecting niche in the plasma membrane, i.e., a bleb niche where the bacteria replicate, and by abrogating vacuolar acidification (Angus et al., 2010; Heimer et al., 2013; Kroken et al., 2018). In neutrophils, ExoS and ExoT reduce bacterial killing by blocking the phagocytic NADPH-oxidase generating reactive oxygen species (Vareechon et al., 2017). However, although *P. aeruginosa* can invade cells and survive in the intracellular environment, the balance between extra and intracellular lifestyle during *P. aeruginosa* pathogenesis remains to be determined.

### Manipulation of sPLA2-IIA by *P. aeruginosa* to eradicate *Staphylococcus aureus*

During airway colonization of CF patients, *P. aeruginosa* not only uses its T3SS to subvert the host immune response to avoid its own eradication but also to eliminate *S. aureus* (Pernet et al., 2014). CF is an autosomal recessive lethal genetic disease characterized by altered bacterial clearance in the airways, leading to recurrent bacterial infections (Strausbaugh and Davis, 2007). However, the bacterial species varies with patient age, with *S. aureus* predominating during childhood and being progressively replaced by *P. aeruginosa* (Registre français de la mucoviscidose – Bilan des données 2020, 2022; Strausbaugh and Davis, 2007).

The secreted group IIA phospholipase A2 (sPLA2-IIA) is a potent bactericidal agent involved in the killing of several Gram-positive bacteria including *S*. *aureus* (Nevalainen et al., 2008). This enzyme is known to selectively kill Gram-positive bacteria by hydrolyzing its membrane phospholipids, leading to bacterial death (Foreman-Wykert et al., 1999) with minimal effects on host cells (Foreman-Wykert et al., 1999). Pernet et al. (2014) showed that infection of bronchial epithelial cells from CF patients by *P. aeruginosa* leads to the induction of sPLA2-IIA production, which in turn results in the killing of *S. aureus* from CF expectorations. However, none of the laboratory or clinical *S. aureus* strains tested were able to induce sPLA2-IIA expression in CF bronchial epithelial cells (Pernet et al., 2014). Induction of sPLA2-IIA expression by *P. aeruginosa* was attributed to the injection of ExoS into epithelial cells, which activates the Krüppel-like factor 2 (KLF2), a transcription factor known to exert anti-inflammatory activities in endothelial cells, monocytes and epithelial cells (O’Grady et al., 2006; Pernet et al., 2014). This mechanism differs from classical pathways known to modulate sPLA2-IIA expression in various cell systems (NF-kB, AP-1, and MAPK) (Menschikowski et al., 2006; Jensen et al., 2009). Nevertheless, whether ExoS induces sPLA2-IIA expression in pulmonary cells other than epithelial cells remains to be examined. This could include alveolar macrophages and endothelial cells, which were shown to be the primary cellular source of sPLA2-IIA in lung tissue (Nevalainen et al., 2008; Hensbergen et al., 2020). Although the mechanism by which ExoS induces KLF2 expression in bronchial epithelial cells is still unclear, the ADPRT domain, and not the GAP domain, is involved in ExoS-induced sPLA2-IIA expression (Pernet et al., 2014). This study supports the notion that *P. aeruginosa* manipulates host cells by inducing their production of sPLA2-IIA which in turn kills *S. aureus* and promotes its establishment in CF airways.

In addition to this subversion mechanism, we have also shown that *P. aeruginosa* down-regulates the expression of the bactericidal antimicrobial peptide (AMP), cathelicidin LL-37, in bronchial epithelial cells *via* a mechanism involving the injection of ExoS into these cells (Abrial et al., 2017). This AMP is known to kill both laboratory and clinical strains of *P. aeruginosa* (Geitani et al., 2019). It was therefore concluded that such a process may allow *P. aeruginosa* to evade the host immune response and initiate infection. Further studies are necessary to identify the signaling pathways involved in LL-37 repression by ExoS.

## Summary

The T3SS is a major player in *P. aeruginosa* pathogenesis. Although T3SS is best known for stimulating the host immune response, this secretion system also allows *P. aeruginosa* to manipulate the inflammatory response to avoid its phagocytosis and to survive intracellularly. T3SS can also manipulate eukaryotic cells to eliminate other pathogens and become the dominant pathogen in certain host organs. Thus, T3SS interactions with host cells contribute to pathogen persistence and could negatively impact the outcome of infection.

The close interactions between T3SS and the immune system suggest that T3SS could be an interesting potential therapeutic target to combat *P. aeruginosa*, and some studies have proposed the use of certain components of T3SS as potential targets for antibacterial drugs and vaccines against *P. aeruginosa* infections (Naito et al., 2018; Moir et al., 2021; Asadi Karam et al., 2022).

## Author contributions

AJ, AS, and LT contributed to the writing of manuscript. All authors contributed to the article and approved the submitted version.

## Acknowledgments

We thank the Fondation Air Liquide for supporting our studies on *P. aeruginosa*. The authors warmly thank Nora Touqui for her precious and meticulous proofreading and English improvement of the present article.

## Conflict of interest

The authors declare that the research was conducted in the absence of any commercial or financial relationships that could be construed as a potential conflict of interest.

## Publisher’s note

All claims expressed in this article are solely those of the authors and do not necessarily represent those of their affiliated organizations, or those of the publisher, the editors and the reviewers. Any product that may be evaluated in this article, or claim that may be made by its manufacturer, is not guaranteed or endorsed by the publisher.
